# The point prevalence of South African male soccer players’ injuries in the Gauteng province

**DOI:** 10.17159/2078-516X/2024/v36i1a17653

**Published:** 2024-08-15

**Authors:** KB De Wet, TJ Ellapen, Y Paul, MP Mahlangu

**Affiliations:** Department of Sport Rehabilitation and Dental Sciences, Faculty of Sciences, Tshwane University of Technology, Pretoria, South Africa

**Keywords:** knee, isokinetic, training history, body composition

## Abstract

**Background:**

The ever-evolving game of soccer is a complex physical contact team sport, exposing its participants to injury.

**Objectives:**

To identify the point prevalence of soccer injuries among young amateur, semi-professional, and professional South African male soccer players.

**Methods:**

The participation of male amateur (n=54), semiprofessional (n=34), and professional (n=57) players provided a cross-sectional overview of the nature of the most predominant types and anatomical sites of injuries affecting soccer players (average age 23.9±4.7 years). All participants completed the Fuller soccer injury questionnaire, ISAK somatotype profiling and knee flexion/extension isokinetic concentric peak torque (Nm) evaluations at 60°/s.

**Results:**

Fifty per cent of the players sustained soccer injuries (*X2*=0.9). Knee (20%) and ankle (19%) were the most vulnerable sites (*X2*=0.00001). Knee-injured players’ right quadriceps torque (199±37 vs 223±38 Nm) and percentage right quadriceps torque relative to body mass (286±54 vs 311 ±39%) was significantly weaker than the non-injured players (p<0.01). The injured players’ right hamstrings/quadriceps (H/Q) torque ratio further significantly differed from the non-injured players’ H/Q torque ratios (79±17 vs 70±9%) (p<0.01).

**Conclusion:**

Male soccer players experience neuromusculoskeletal injuries, with their knees and ankles being the most vulnerable. Knee-injured players had weaker quadriceps isokinetic strength than non-injured players.

Soccer is a multifaceted physical team sport, exposing participants to injury during the games and/or practice. ^[1]^ The modern soccer game has evolved to be played at a quicker and more aggressive pace. ^[1,2,3]^ Predisposing risk factors of soccer injuries include age, conditioning status, training load, and competition level of play. ^[1,2,3]^ Young soccer players (aged 12 – 19 years) must be conditioned to become resilient to succeed in the professional soccer arena. ^[4]^ Young players must engage in strenuous physical and skills conditioning programmes to attain this end. ^[4]^ The frequency of soccer injuries inhibits players’ and team performance and success. ^[4]^ Researchers must monitor the type and nature of soccer injuries since the modern game has evolved. Medical support staff managing soccer players need to be abreast of the latest injury statistics, trends, and potential strategies to combat these inevitable soccer injuries. ^[3]^

Soccer entails intermittent running at high velocity, explosive sprinting and jumping, and rapid change of direction contributing to musculoskeletal injuries. ^[5]^ Ankle and knee injuries are prevalent in the game of soccer. ^[6, 7]^ O’Donnell and colleagues reported that approximately 24% of all soccer injuries occur at the knee joint. ^[5]^ The causes of knee injuries include rapid changes in direction at high velocity, jumping, and poor landing. ^[5]^ Rapid change in direction at high velocity and poor landing can be dangerous, as these movements produce large volumes of torques, stressing and destabilising the surrounding knee muscles, ligaments, and soft tissue structures. ^[5]^ The hamstring quadriceps ratio (H/Q) is a strong indicator of knee muscle imbalances and potential risk of injury. ^[5]^ The H/Q ratio is assessed to determine the hamstrings and quadriceps moment-velocity patterns, which can be used to evaluate knee functional ability and hamstring quadriceps muscle imbalance. ^[5]^ O’Donnell et al. and Croisier et al. reported that an H/Q ratio of less than 55% was associated with an increased risk of knee injury. ^[5,8]^ Grooms et al documented a significant reduction in in-season knee injuries due to preseason conditioning aimed at amending H/Q ratio imbalances. ^[9]^ However, there are a limited number of studies conducted among South African soccer players to confirm this injury pathomechanism. This study reviewed the injury profile, training history and the H/Q isokinetic concentric torque ratio of young amateur, semi-professional, and professional soccer players to ascertain whether South African knee-injured players also portray similar trends.

## Methods

### Study design

One hundred and forty-five male soccer players participated in a cross-sectional observational injury and isokinetic strength enquiry by voluntary informed consent. Ethical approval was obtained from the Tshwane University of Technology Research Committee (FCRE 2022/08/001 (SCI) (FCPS 02). Participants were affiliated with soccer teams of the Gauteng province in South Africa. The participation of amateur (n=54), semiprofessional (n=34) and professional (n=57) players provided a cross-sectional overview of the nature of the most predominant muscular, ligamentous, skeletal, and nerve injuries affecting soccer players. A professional soccer player is an athlete whose primary occupation is to play soccer. These athletes undergo physical training, practice with their affiliated team, and compete professionally in a football league. Semi-professional soccer players are athletes who do not participate on a full-time basis but still receive some payment. These players’ primary occupation is some employment other than as a soccer player. Semi-professional soccer players receive regular payment from their affiliated team, which is generally at a lower rate than a professional soccer player. They undergo physical training and practice with their team and players in a league. Amateur soccer player refers to any player registered to play or who intends to be registered for a soccer club in a soccer league. Generally, amateur players do not receive a financial stipend for soccer participation.

Gatekeepers’ approval was secured from the respective clubs, followed by a briefing session to explain the nature of the study. At the briefing sessions, players, coaching staff, and support staff received an information letter (describing the study) and informed consent from participants. An opportunity was provided for attendants to ask questions, which were answered by the researcher. A date was scheduled to collect the data after gathering the informed consent. The Fuller injury questionnaire was used to collect players’ soccer injury history. ^[10]^ The authors adopted a six-month point prevalence soccer injury history (April–September 2023). The kinanthropometrical measures were recorded: body mass, stature, per cent fat mass, and per cent muscle mass according to International Society for the Advancement of Kinanthropometry protocol. ^[11]^ Concentric isokinetic knee flexion and extension torque measures (Nm) at 60°/s were recorded utilising a HUMAC NORM dynamometer (CSMi; Stoughton) implementing the Sole et al. protocol (ICC=0.9). ^[12]^ The inclusion criteria entailed that players were male, participating in a formal South African soccer league structure in the Gauteng region (either in a professional, semiprofessional, and/or amateur capacity), aged 18–35 years old, and voluntarily participating.

### Statistical analysis

All data were analysed using the Statistical Package for Social Sciences (SPSS) version 25.0 for Windows (SPSS Inc, Chicago, IL, USA). Descriptive statistical analyses involved mean, standard deviation, and percentage changes. Inferential statistical analyses involved chisquare (*X2*), t-test and effect size. Alpha was set at p<0.05.

## Results

### Demographic characteristics

The injured players’ age, body mass, stature, and percentage muscle mass significantly differed from the non-injured players (p<0.005) (Table 1). The injured players were younger, with a lighter body mass and body mass index, and possessed less percentage muscle mass (Table 1).

### Training history

The training history profile of the non-injured players differed from the injured players in regard to the average duration of a training session per week and the frequency of aerobic training practice (p<0.05). Aerobic training involved running. The injured players’ average training session duration was significantly longer than the non-injured players (p=0.04) (Table 2). The non-injured players performed more aerobic training per week than the injured players (p=0.001) (Table 2).

### General injury profile analyses

There were 73 injured (50%) of the 145 participants (*X2* =0.9). The point prevalence injury rate for the six months prior to the evaluation date was 0.000365 per player. Most vulnerable anatomical sites of injury included knee (27%), ankle (26%), thigh (23%), shoulder (5.4%), lower back (5.4%), lower limb (4.1%), groin (2.7%), hand (1.3%), buttock (1.3%), foot (1.3%), and head (1.3%) (*X2*=0.00001) (Table 3). Type of injury sustained by the participants included muscle injury (29%), joint injury (27%), ligament injury (13%), nerve injury (3.4%), bone injury (1.7%) and unexplained injury (24%) (*X2*=0.0001). Number of day/s participants missed practice and/or games: one day (10 participants), two days (11 participants), three days (1 participant), four days (3 participants), five days (9 participants), more than five days (24 participants) (*X2*=0.0008). The choice of healthcare practitioners that the injured participants consulted when sustaining their injury included physiotherapists (26 participants), general practitioners (17 participants), orthopaedic surgeons (10 participants), biokineticists (7 participants), massage therapists (3 participants), chiropractor therapist (0) and others not identified (one participant) (*X2* =0.000001). Fifty participants (68%) of the 73 injured players practised and/or played through the injury (*X2* =0.0009). Of the 50 injured participants who practised and/or played through the injury, 22 participants’ (44%) pain symptoms increased (*X2* =0.3). Of the 17 goalkeepers, seven were injured (41%), 27 of 40 defenders were injured (67%), 31 of 72 mid-fielders were injured (43%), and eight out of 16 forwards were injured (50%) (Table 3).

### Comparative isokinetic knee strength analyses of knee injured players and non-injured knee players

Injured players’ right quadriceps strength and percentage of right quadriceps strength relative to the player’s body mass was significantly weaker than the non-injured players (p<0.01) (Table 4). The injured players’ right hamstrings/quadriceps (H/Q) torque ratio significantly differed from the non-injured players (p<0.01) (Table 4). The injured H/Q ratio varied greater from the normal ratio, where the non-injured players were closer to the normal ratio. Seventeen (85%) of the twenty knee-injured players were right-leg dominant (X2 =0.001).

## Discussion

The study identified that 50% of the cohort were injured within the six-month period during matches and/or practice, which concurs with previous studies documenting the high volume of injuries players sustain during a soccer season. ^[1,3,13]^ This study did find the injured players were younger, weighed less, were shorter, and possessed less muscle mass than their non-injured counterparts, which differed from previous studies. ^[15,16]^ Hagglund and colleagues reported that players’ body mass and stature are not predisposing risk factors to soccer injuries. ^[15]^ Faude et al. recommended that injury risk should be assessed on an individual basis. ^[16]^ These findings necessitate further empirical investigation to confirm their reliability.

Muscle strains, bone fractures and ligament sprains were the most common types of injuries cited by players, which corresponds with Patel et al. ^[13]^ The most vulnerable anatomical sites of injury were the knee and ankle, which is supported by findings of Masenya and Onaka et al. ^[6,7]^ This study found that knee injured players’ right quadriceps strength was significantly weaker than non-injured players. Hannon et al. reported that quadriceps muscle strength weakness predisposes athletes to knee joint injury. ^[17]^ The knee-injured players’ H/Q torque ratio differed from non-injured players. Coombs reported that the normal H/Q concentric isokinetic strength ratio is approximately 0.6, which is frequently used as an injury prevention and rehabilitation yardstick. ^[18]^ O’Donnell described deviant H/Q strength ratios is a risk factor for knee and thigh injuries. ^[5]^ In 85% of the knee-injury players, the dominant leg (knee) was injured. De Lang and colleagues reported that soccer players demonstrated a 1.6 times greater risk of injury to the dominant limb due to overuse. ^[19]^ Soccer players use their dominant limbs to kick and pass the ball at high velocities with greater frequency than the non-dominant limbs. ^[19]^

### Limitations

The current sample size impacts the statistical robustness of the study. Empirical investigations with a bigger sample size and continuous monitoring is proposed to increase the significance of the findings. The age range of the sample varied from 18 to 35 years old, which included three growth stages. It would be advisable to examine more homogenous age stratums of different age groups, as the players would be in a single growth stage.

## Conclusion

Male soccer players experience numerous neuromusculoskeletal injuries within a season. This study determined that the injured players were younger, weighed less, were shorter, and possessed less muscle mass than their non-injured counterparts. The average duration of training sessions for injured players was significantly longer than that of non-injured players. The knee and ankle joints were the most vulnerable anatomical sites to injury. Knee-injured players had weaker quadricep isokinetic strength than non-injured players. Eighty-five per cent of the knee injuries were sustained on players’ dominant limb. This study’s findings were constructed in reviewing the comparative analysis of knee injury versus non-injured isokinetic concentric extension and flexion torque of the cohort. There could have been other predisposing risks contributing to the prevalent knee injuries, which warrant further investigation in prospective empirical studies. In light of these findings, it could be concluded that players, strength and conditioning scientists, and soccer support staff consider these findings and amend their conditioning programmes to improve the physical conditioning profiles of players to reduce the risks of injury and ensure they compete safely.

## Figures and Tables

**Table 1:** Comparison of the demographic characteristics of injured and non-injured players

Variables	Injured (n=73)	Non-injured (n=72)	p-value	Effect size

Age (years)	23.0 ± 3.7	24.6 ± 5.0	0.003*	3.0
Body mass (kg)	68.2 ± 8.2	74.8 ± 9.5	0.0001*	0.7
Stature (m)	173.3 ± 7.7	177.0 ± 6.8	0.002*	0.5
Fat mass (%)	9.0 ± 2.4	9.0 ± 2.1	0.9	0.0
Muscle mass (%)	42.3 ± 2.5	43.2 ± 2.0	0.02*	0.4

* indicates statistical significance (p<0.05).

**Table 2:** Training history of injured and non-injured players

Training histroy	Injured (n=73)	Non-injured (n=72)	p-value	Effect size

Years affiliated to club	2 ± 2	2 ± 2	0.57	0.05
Number of months training in a year	9 ± 1	10 ± 1	0.4	0.6
Number of training sessions/week	4 ± 1	5 ± 1	0.27	0.8
Average duration of training session/week (minutes)	99 ± 34	82 ± 17	0.04*	−0.6
Strength training (sessions/week)	1 ± 1	1 ± 1	0.65	−0.2
Aerobic training (sessions/week)	2 ± 2	3 ± 1	0.001*	1.0
Flexibility training (sessions/week)	2 ± 2	2 ± 2	0.29	0.1

* indicates statistical significance (p<0.05).

**Table 3:** Anatomical sites of injury stratified according to playing positions (n=73)

Anatomical sites of injury	Goal keepers (n=7)	Defenders (n=27)	Midfielders (n=31)	Forwards (n=8)	Total number of injuries

Neck	0	0	0	0	0
Shoulders	1	3	0	0	4
Elbow	0	0	0	0	0
Forearm	0	0	0	0	0
Hand	1	0	0	0	1
Middle back	0	0	0	0	0
Lower back	0	2	1	1	4
Buttock	0	0	1	0	1
Thigh	1	6	7	3	17
Knee	2	3	13	2	20
Lower limb	1	2	0	0	3
Ankle	1	10	6	2	19
Foot	0	0	1	0	1
Others	0	1	2	0	3

**Table 4:** Comparative review of knee-injured and non-injured players isokinetic concentric extension and flexion torque at 60°/s

Isokinetic measures	Injured participants (n=20)	Non-injured players (n=125)	p-value	Effect size

Right quadriceps extension torque (Nm)	199 ± 37	223 ± 38	0.01*	0.6
Left quadriceps extension torque (Nm)	199 ± 39	212 ± 34	0.17	0.3
Percentage of right quadriceps extension torques relative to body mass (%)	286 ± 54	311 ± 39	0.05*	0.6
Percentage of left quadriceps extension torques relative to body mass (%)	282 ± 55	296 ± 37	0.2	0.1
Right hamstrings flexion torque (Nm)	156 ± 32	155 ± 26	0.9	0.00
Left hamstrings flexion torque (Nm)	147 ± 29	150 ± 26	0.6	0.1
Percentage of right hamstrings torques relative to body mass (%)	222 ± 31	217 ± 26	0.4	−0.1
Percentage of left hamstrings torques relative to body mass (%)	210 ± 30	210 ± 26	0.9	−0.00
Right hamstrings/quadriceps torque ratio	79 ± 17	70 ± 9	0.02*	−0.8
Left hamstrings/quadriceps torque ratio	75 ± 15	71 ± 9	0.2	−0.3

* indicates statistical significance (p<0.05). Nm, newton-metre extension torque measures.
